# Gut permeability is associated with hypertension and measures of obesity but not with Endothelial Dysfunction in South African youth

**DOI:** 10.4314/ahs.v21i3.26

**Published:** 2021-09

**Authors:** Ezona E Ntlahla, Mvuyisi MO Mfengu, Godwill A Engwa, Benedicta N Nkeh-Chungag, Constance R Sewani-Rusike

**Affiliations:** 1 Department of Human Biology, Faculty of Health Sciences, Walter Sisulu University PBX1, 5117, Mthatha, South Africa; 2 Department of Biological and Environmental Sciences, Faculty of Natural Sciences, Walter Sisulu University PBX1, 5117, Mthatha, South Africa

**Keywords:** Gut permeability, hypertension, obesity, endothelial dysfunction, inflammation

## Abstract

**Background:**

Though gut permeability has shown to be associated with measures of obesity and hypertension, its relationship with endothelial dysfunction, an early predictor for cardiovascular diseases remains unknown.

**Objective:**

This study assessed the relationship between hypertension, measures of obesity, gut permeability and endothelial dysfunction.

**Methods:**

A cross-sectional quantitative study which enrolled 151 South African youths was conducted. Anthropometric and blood pressure measurements were performed. Zonulin, a marker for gut permeability; adiponectin, an anti-inflammatory molecule, as well as asymmetric dimethylarginine (ADMA) and Nitric oxide (NO) which are markers for endothelialfunction were assayed.

**Results:**

Approximately eighteen percent (17.88%) of the participants were hypertensive while 40.4% were pre-hypertensive. Adiponectin significantly increased in hypertensive subjects and negatively correlated (p<0.05) with measures of obesity but was not associated with gut permeability and endothelial dysfunction. Increased body mass index (BMI) and visceral fat (VF) predicted reduced adiponectin (inflammation). Zonulin was significantly higher (p<0.05) in hypertensive subjects and positively associated (p<0.05) with systolic blood pressure (SBP) in females. A positive relationship (p<0.05) was observed between zonulin and measurements of obesity. Moreover, zonulin negatively associated (p<0.05) with ADMA but positively associated (p<0.05) with NO in males. Increased VF and waist circumference predicted gut permeability.

**Conclusion:**

Gut permeability was associated with hypertension and measures of obesity but not with markers of endothelial dysfunction in a South African youth population.

## Introduction

Increased intestinal permeability has recently been proposed to be an integral element in the pathogenesis of obesity and metabolic syndrome inclusive of hypertension^1^–^3^. Increased gut permeability, also known as hyper-permeable intestines or “leaky gut” is a condition whereby disassembly of tight junction protein results in increased porosity of intestinal paracellular pathway^4^,^5^. The tight junctions are made up of transmembrane proteins which include claudins, occludins, angulins, tricellulin and junctional adhesion molecules (JAM) 6–8. These proteins interact between themselves and with intracellular scaffolding proteins including zonula occludens (ZOs) which are found anchored in the cytoskeleton of the cell. Interaction between these proteins and ZOs anchor maintain the integrity of the tight junction and also control the passage of molecules across the intestine^9^. Disruption of this barrier results in increased intestinal permeability and leakage of gut bacteria and bacterial products into the blood stream. Circulating bacterial products such as lipopolysaccharide (LPS) stimulate the synthesis of zonulin from the intestine and liver thus increasing its concentration in blood^9^. As such, zonulin is considered the main physiological regulator of mucosal permeability and biomarker for gut permeability since it is capable of causing tight junction disassembly^10^. Seepage of bacterial substances into the circulation results in immune cell recruitment and activation leading to the release of pro-inflammatory cytokines such as interleukin (IL)-1B, IL-12, IL-6 and TNF-α, all of which promote and accelerate systemic inflammation^9^.

Inflammation is accompanied by oxidative stress, uncoupling of endothelial nitric oxide synthase (eNOS), reduced nitric (NO) bioavailability and reduced adiponectin, all of which contribute to endothelial dysfunction 11–13. Adiponectin is known to possess vasculoprotective effects via its anti-obesity and anti-inflammatory effects but it remains unknown how it is affected by gut permeability. Hypoadiponectinaemia is considered an independent risk factor of endothelial dysfunction and hypertension^14^. Reduced plasma adiponectin promotes the synthesis of arginase, an enzyme in the vasculature that inhibits the production of NO by competing with eNOS for the substrate L-arginine^15^, ^16^, thereby leading to endothelial dysfunction which is an early predictor of cardiovascular diseases.**27** Additionally, asymmetric dimethylarginine (ADMA) is an endogenous inhibitor of eNOS^18^ and is increased in conditions associated with endothelial dysfunction such as dyslipidaemia, 19 insulin resistance,^20^ diabetes mellitus,^21^ and hypertension^22^, ^23^. As such, ADMA is now used as a marker for endothelial dysfunction and a risk factor for cardiovascular disease^24^.

Several factors such as altered gastrointestinal tract peristalsis, antibiotic use, radiation, psychological and physical stress, and dietary changes,^25^ as well as obesity^26^ are known causes of gut permeability. As explained above, gut permeability may contribute to endothelial dysfunction and hypertension through systemic inflammation and oxidative stress. Additionally, it is postulated that hypertension may also be a factor that promotes and exacerbates gut permeability. A previous study suggested that increased sympathetic drive which plays a central role in hypertension is associated with increased gut wall permeability and increased inflammatory status^27^. Another study demonstrated a link between gut microbiota dysbiosis and increased gut permeability and inflammation in patients with pulmonary arterial hypertension^28^. Altogether, these and other studies support an integrated role played by obesity, gut permeability and endothelial dysfunction in the pathogenesis of hypertension.

Hypertension is a global health issue that contributes to the burden of cardiovascular diseases (CVDs). Approximately 1.13 billion people worldwide are hypertensive, with about two-thirds living in low- and middle-income countries^29^ and it is projected that 1.5 billion adults will be hypertensive in the next coming years^30^. The increasing prevalence of hypertension especially in developing countries like South Africa is of concern. In 2017, about 35.1% of South Africans were hypertensive^31^ while in the Eastern Cape province of South Africa hypertension is the second leading cause of death after diabetes^32^. With this increasing prevalence of hypertension, it is imperative to investigate the contribution of gut health in the form of gut permeability as well as endothelial dysfunction in this population. Though previous studies have shown a positive association between zonulin, blood pressure and measures of obesity,^33^, ^34^ such findings are yet to be established in an African population of young, apparently healthy adults. More so, the relationship between gut permeability and endothelial dysfunction has not been addressed. Hence, this study was aimed to assess the relationship between hypertension, measures of obesity, gut permeability and endothelial dysfunction in a South African population.

## Materials and methods

### Study design and population

This was a cross-sectional quantitative study which enrolled 151 (77 females and 74 males) apparently healthy students age 18–25-year-old at Walter Sisulu University, Mthatha, Eastern Cape Province of South Africa.

### Ethical consideration

The study was conducted in accordance with the guidelines of the Helsinki Declaration (2008 reviewed version) as well as local and national regulations in South Africa. Ethical approval was obtained from the Faculty of Health Sciences Research Ethics Committee of Walter Sisulu University with approval number: [051/2019] on the 26 of June 2019. After careful explanation of the purpose and aim of the study, written informed consent was obtained from each volunteering student before enrolment into the study. The study adhered to the standards of reporting and the identity of the participants was kept confidential in accordance with the National Data Protection Acts. There were no important changes in the methods after study commencement.

### Inclusion/Exclusion criteria

Registered students both male and female of Walter Sisulu University aged 18–25 years were recruited for the study. Students with any history of allergies to gluten or on a gluten-free diet, pregnant, lactating or on medications such as antidepressants, caffeine or hormonal birth control pills were excluded from the study.

### Anthropometric measurements

Anthropometric measurements were performed in accordance with the International Standards for Anthropometric Assessments^35^ on all participants. Participants' waist circumference (WC) was measured at the approximate mid-point between the lower margin of the last palpable rib and the top of the iliac crest using an anthropometric tape in centimetres (cm). Height was measured using a wall-mounted stadiometer (Electronic body scale TCS-200-RT, Ningbo, China) and recorded to the nearest 0.1cm. Body composition was assessed using the Omron bioimpedance scale (Omron BF500, Omron Healthcare Inc., Illinois, USA) which measured weight (kg), body mass index (BMI) in kg/m^2^, visceral fat (VF%) and skeletal muscle fat (SMF%).

### Blood pressure measurements

Participants were asked to sit in an upright position and remove any clothing that was tight on their left arm. After resting for at least 10 minutes, a cuff connected to an automatic Omron M3 sphygmomanometer (HBP-1100; Omron Healthcare Co. Ltd., Illinois, USA) was placed around the participants left arm and was switched on to measure their systolic blood pressure (SBP) and diastolic blood pressure (SBP) in millimetres mercury (mm Hg) and heart rate (bpm). To ensure accuracy of the readings, three measurements were taken, the average determined and classified as hypertension when SBP ≥140mmHg and/or DBP≥ 90 mmHg, pre-hypertension when SBP is between 120 and 139 mmHg and/or DBP is between 80 and 90 mmHg while SBP< 120 and DBP<80 was considered as normal^36^.

### Blood collection and biochemical analysis

Five millilitres (5ml) of blood was collected into ethylenediaminetetraacetic acid (EDTA) tubes and was centrifuged (Eppendorf 5810 R) for 15 minutes at 1000×g to obtain plasma. The plasma was used to quantify ADMA and NO which are markers for endothelial function, zonulin, a marker for gut permeability and adiponectin. Zonulin, ADMA and adiponectin were measured using enzyme-linked immunosorbent assay (ELISA) kits (Elabscience® USA with catalogues E-EL-H5560, E-EL-0042, E-CL-H0004 respectively) according to manufacturers' protocol. Griess reagent was used to measure NO indirectly as nitrite in plasma as previously described^37^.

### Statistical analyses

Data was analysed using GraphPad Prism version 5. The data was presented as mean ± standard error of the mean (S.E.M). Analysis of variance (ANOVA) was used to compare mean differences of continuous variables between normotensive, pre-hypertensive and hypertensive subjects. Two-way ANOVA was used to compare mean differences of continuous variables between males and females. Pearson correlation was used to determine the relationship of gut permeability with measures of endothelial function, obesity, and blood pressure. Multivariate linear regression analysis was done to assess predictors of inflammation, gut permeability and endothelial function. A p-value ≤0.05 was considered to be statistically significant.

## Results

### Prevalence of pre-hypertension and hypertension

Among the 151 study participants, approximately eighteen percent (17.88%) were hypertensive while 40.4% were pre-hypertensive. The prevalence of hypertension was higher in males (21.62%) than in females (14.29%). A similar trend was observed for pre-hypertension as the prevalence was higher in males (48.64%) than females (32.46%). Results are summarised in Table 1.

**Table 1:** Prevalence of pre-hypertension and hypertension

Gender	Population size	NT	Pre-HPT	HPT
	n	n (%)	n (%)	n (%)
**Female**	77	41 (53.25)	25 (32.46)	11 (14.29)
**Male**	74	28 (37.84)	36(48.64)	16 (21.62)
**Cohort**	151	69 (45.60)	61 (40.40)	27 (17.88)

**Note:** n: number of participants; NT: Normotensive; Pre-HPT; Pre-hypertensive; HPT: Hypertensive

### Population characteristics

The study population was grouped as hypertensive and pre-hypertensive, and compared to normotensive subjects. Anthropometric and obesity measures including weight, BMI, WC and VF% were significantly (p<0.05) higher in hypertensive subjects compared to pre-hypertensive and normotensive subjects. Similarly, the SBP and DBP were significantly (p<0.001) higher in hypertensive and pre-hypertensive participants compared to normotensive participants (Table 2).

**Table 2:** Characteristics of participants based on blood pressure

Characteristics	NT	Pre-HPT	HPT
	(n=63)	(n=61)	(n=27)
**Age**	21.9±0.3	22.1±0.2	22.2±0.3
**Weight (kg)**	63.7±1.6	66.9±2.1	74.9±3.5**
**Height (m)**	1.63±0.01	1.66±0.01	1.66±0.01
**BMI (Kg/m^2^)**	23.9±0.6	24.1±0.7	27.1±1.2*
**VF (%)**	4.6±0.3	5.7±0.4	8.0±1.1***
**SMF (%)**	29.0±1.2	29.7±1.1	30.9±1.9
**WC (cm)**	78.3±1.5	77.6±1.8	86.0±2.9*
**DBP (mmHg)**	72.1±0.7	79.9±0.8***	95.1±1.7***
**SBP (mmHg)**	108.7±0.9	123±1.0***	141.9±2.2***
**HR (bpm)**	73.5±1.5	72.4±1.6	74.5±2.4

**Note:** Data was presented as mean ± S.E.M; NT: Normotensive; Pre-HPT: Pre hypertensive; HPT: Hypertensive; BMI: Body Mass Index; VF: Visceral fat; SMF: Skeletal muscle fat; WC: Waist circumference; DBP: Diastolic blood pressure; SBP: Systolic blood pressure; HR: Heart rate

* *p*<0.05

** *p*<0.01

*** *p*<0.001 compared to NT

### Effect of hypertension on endothelial function markers

**Figure 1:**
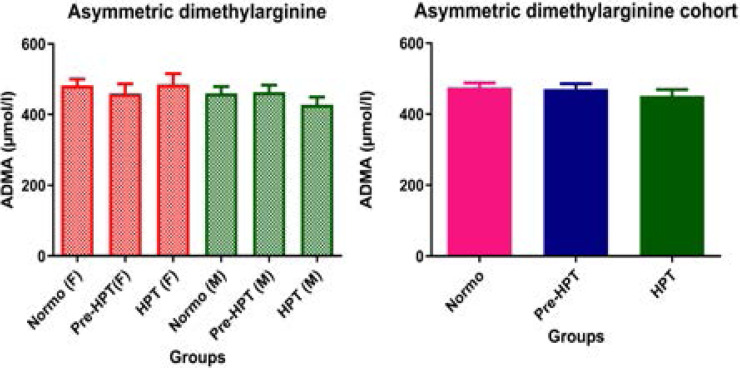
Comparison of ADMA in hypertensive, pre-hypertensive and normotensive participants. Data was presented as mean± S.E.M. ADMA: Asymmetric dimethylarginine; NT: Normotensive; Pre-HPT: Prehypertensive; HPT: Hypertensive; F: Females; M: Males;

**Figure 2:**
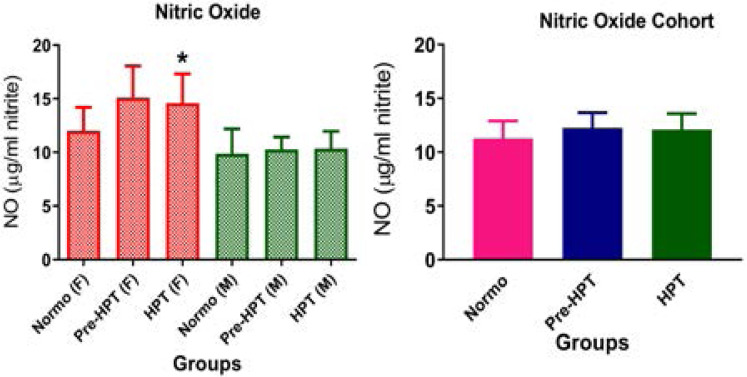
Comparison of nitric oxide in hypertensive, pre-hypertensive and normotensive participants. Data was presented as mean± S.E.M. NO: Nitric oxide; NT: Normotensive; Pre-HPT: Pre-hypertensive; HPT: Hypertensive; F: Females; M: Males; *p<0.05

The results of this study showed that ADMA concentration was similar in all groups and thus, showed no significant (p>0.05) difference between hypertensive, pre-hypertensive and normotensive participants in the cohort population as well as among males and females (Figure 1). Moreover, though NO was not significantly (p>0.05) different between hypertensive, pre-hypertensive and normotensive participants in the cohort as well as among males, it was significantly higher (p<0.05) in hypertensive females than in normotensive and pre-hypertensive females (Figure 2).

### Effect of hypertension on gut permeability

**Figure 3:**
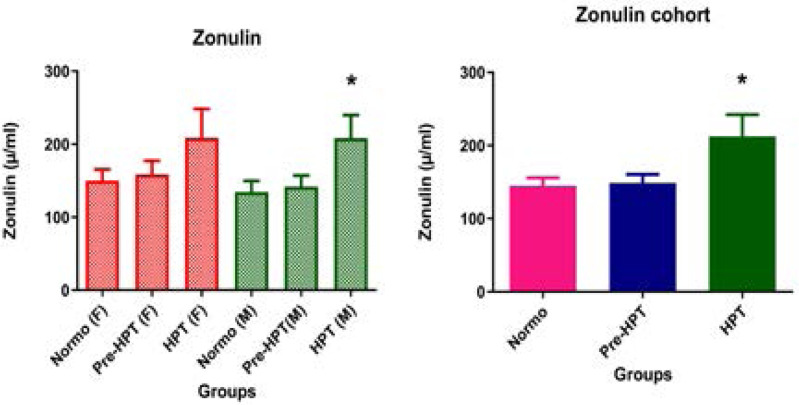
Comparison of plasma zonulin concentration in hypertensive, pre-hypertensive and normotensive participants. Data was presented as mean± S.E.M. NT: Normotensive; Pre-HPT: Pre hypertensive; HPT: Hypertensive; F: Females; M: Males; *p<0.05 compared to NT.

Zonulin concentration was significantly (p<0.05) higher in hypertensive participants in the cohort as well as in males compared to normotensive and pre-hypertensive participants. Though zonulin concentration was not significantly (p=0.055) different in hypertensive females, it showed a trend towards a higher level compared to normotensive and pre-hypertensive females (Figure 3).

### Effect of hypertension on plasma adiponectin

**Figure 4:**
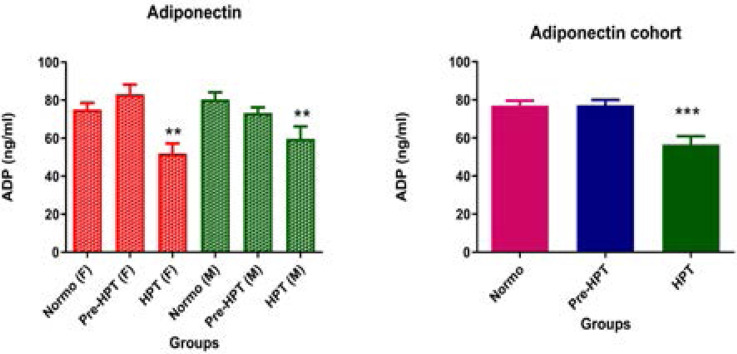
Comparison of plasma adiponectin (ADP) concentration in hypertensive, pre-hypertensive and normotensive participants. Data was presented as mean± S.E.M. NT: Normotensive; Pre-HPT: Pre hypertensive; HPT: Hypertensive; F: Females; M: Males; **p<0.01; ***p<0.001 compared to NT.

Adiponectin (ADP) was significantly (p<0.001) lower in hypertensive compared to pre-hypertensive and normotensive participants in the cohort. Similarly, both hypertensive males and females had a significantly (p<0.01) lower lever of adiponectin compared to their pre-hypertensive and normotensive counterparts (Figure 4).

### Relationship of adiponectin with blood pressure, endothelial function and body composition

Adiponectin (ADP) negatively correlated (p<0.001) with SBP in the cohort and in males and females, and also with DBP in the cohort and in males. Similarly, adiponectin negatively correlated (p<0.001) with measures of body composition (VF and BMI) in the cohort and in males and females as well as with WC in the cohort and females (Table 3).

**Table 3:** Correlation of adiponectin with blood pressure, endothelial function and body composition

Variable ADP vs.	Females		Males		Cohort	

	*r*	*p*-value	*r*	*p*-value	*r*	p-value
**DBP**	-0.20	0.08	-0.22	**0.05**	-0.21	**0.01**
**SBP**	-0.28	**0.01**	-0.41	**0.0003**	-0.33	**<0.0001**
**ADMA**	0.16	0.17	0.02	0.84	0.09	0.26
**NO**	-0.04	0.72	0.03	0.80	-0.02	0.85
**Zonulin**	-0.20	0.09	0.06	0.59	-0.07	0.37
**WC**	-0.39	**0.0004**	-0.19	0.11	-0.31	**<0.0001**
**VF**	-0.39	**0.0004**	-0.35	**0.002**	-0.36	**<0.0001**
**BMI**	-0.44	**<0.0001**	-0.36	**0.002**	-0.4	**<0.0001**

**Note:** *r*: correlation coefficient; DBP: Diastolic blood pressure; SBP: Systolic blood pressure; ADMA: Asymmetrical dimethylarginine; NO: Nitric oxide; ADP: Adiponectin BMI: Body Mass Index; VF: Visceral fat; WC: Waist circumference

### Relationship of endothelial function, measures of obesity and blood pressure with gut permeability

Zonulin positively correlated (p<0.05) with SBP in females. A significant (p<0.05) negative correlation was observed between zonulin and ADMA in the cohort as well as in males and females while a significant (p<0.05) positive correlation was observed between zonulin and NO in males and study cohort. A positive correlation p<0.05) was also found between zonulin and anthropometric measurements (WC, BMI and VF) in females and cohort while there was no correlation (p>0.05) between anthropometric measurements in males. There was no correlation (p>0.05) between zonulin and adiponectin in the cohort as well as in males and females (Table 4).

**Table 4:** Correlation of gut permeability with blood pressure, endothelial function, adiponectin and body composition

Variable: Zonulin vs.	Females		Males		Cohort	

	*r*	*p*-value	*r*	*p*-value	*r*	*p*-value
**DBP**	0.10	0.40	0.08	0.5	0.08	0.3
**SBP**	0.22	**0.05**	0.06	0.61	-0.09	0.29
**ADMA**	-0.28	**0.01**	-0.40	**0.0004**	-0.33	**<0.0001**
**NO**	0.19	0.09	0.37	**0.0013**	0.26	**0.001**
**ADP**	-0.02	0.09	0.06	0.59	-0.07	0.37
**WC**	0.29	**0.01**	0.06	0.61	0.14	0.09
**VF**	0.48	**<0.0001**	-0.02	0.84	0.20	**0.01**
**BMI**	0.38	**0.0007**	-0.02	0.84	0.20	**0.01**

**Note:** *r*: correlation coefficient; DBP: Diastolic blood pressure; SBP: Systolic blood pressure; ADMA: Asymmetrical dimethylarginine; NO: Nitric oxide; ADP: Adiponectin BMI: Body Mass Index; VF%: Visceral fat; WC: Waist circumference.

### Predictors of inflammation, gut permeability and endothelial dysfunction

Adjusted linear regression analysis of a fitted model (F=26.012; p<0.001) for the relationship between adiponectin with measures of obesity, gut permeability and endothelial function showed increased visceral fat to predict inflammation; that is, reduced adiponectin (R2= 0.15, Adj.R2= 0.145; p<0.001) in the cohort. In males, a fitted model (F=16.086; p<0.001) showed increased BMI to predict inflammation (R2= 0.183, Adj. R2= 0.171; p<0.001) while in females, a fitted model (F=9.766; p<0.001) showed increased visceral fat to predict inflammation (R2= 0.213, Adj.R2= 0.192; p<0.001). Results are summarized in Table 5.

**Table 5:** Regression analysis of adiponectin with blood pressure, endothelial function and body composition

Variable ADP vs.	Females		Males		Cohort	

	*B*	*p*-value	*B*	*p*-value	*B*	p-value
**DBP**	-0.044	0.688	-0.017	0.877	-0.029	0.706
**SBP**	-0.145	0.179	0.095	0.378	-0.040	0.602
**ADMA**	-0.103	0.355	0.161	0.131	0.020	0.795
**NO**	0.112	0.305	0.075	0.487	0.073	0.338
**Zonulin**	-0.016	0.895	-0.004	0.973	-0.040	0.599
**WC**	-0.157	0.287	0.109	0.470	-0.098	0.315
**VF**	-4.452	**<0.001**	-0.157	0.621	-2.845	**<0.001**
**BMI**	-0.078	0.646	-2.044	**<0.001**	-0.142	0.198

**Note:** *B*: regression coefficient; DBP: Diastolic blood pressure; SBP: Systolic blood pressure; ADMA: Asymmetrical dimethylarginine; NO: Nitric oxide; ADP: Adiponectin BMI: Body Mass Index; VF: Visceral fat; WC: Waist circumference.

Also, adjusted linear regression analysis of a fitted model (F=4.212; p=0.017) for the relationship between zonulin with measures of obesity, inflammation and endothelial function showed reduced DBP and increased waist circumference to predict gut permeability (R2= 0.055, Adj.R2= 0.042; p<0.05) in the cohort. In females, a fitted model (F=9.469; p<0.001) showed reduced DBP and increased visceral fat to predict gut permeability (R2= 0.208, Adj.R2= 0.186; p<0.05) in females. However, in males, an unfitted model (F=0.78, p=0.677) showed no parameter to predict gut permeability (R2= 0.145, Adj.R2= -0.041; p>0.05). Results are summarised in Table 6.

**Table 6:** Regression of gut permeability with blood pressure, endothelial function, adiponectin and body composition

Variable: Zonulin vs.	Females		Males		Cohort	

	*B*	*p*-value	*B*	*p*-value	*B*	*p*-value
**DBP**	-1.865	**0.047**	-3.39	0.087	-1.778	**0.032**
**SBP**	0.086	0.543	1.516	0.241	0.154	0.154
**ADMA**	-0.096	0.378	0.021	0.863	-0.005	0.954
**NO**	-0.176	0.095	0.662	0.721	-0.016	0.841
**ADP**	-0.026	0.818	-0.566	0.488	-0.080	0.327
**WC**	0.074	0.606	0.630	0.733	1.239	**0.049**
**VF**	17.544	**<0.001**	-7.46	0.533	0.090	0.268
**BMI**	-0.071	0.670	4.174	0.777	0.133	0.102

**Note:** *B*: Regression coefficient; DBP: Diastolic blood pressure; SBP: Systolic blood pressure; ADMA: Asymmetrical dimethylarginine; NO: Nitric oxide; ADP: Adiponectin BMI: Body Mass Index; VF%: Visceral fat; WC: Waist circumference.

## Discussion

Hypertension is a non-communicable disease which is associated with high morbidity and mortality. It is a silent threat to the health of people all over the world, with over one-third of the world's population affected^38^. Developing countries such as South Africa are faced with a growing burden of non-communicable and communicable diseases^39^. In this present study, 17.88% of the study population were hypertensive while 40.4% were pre-hypertensive. This finding is in line with a previous study in South Africa which showed high prevalence of hypertension as well as pre-hypertension^40^. The prevalence of pre-hypertension and hypertension were higher compared to a cohort study in learners <18-year-old in KwaZulu-Natal, South Africa, which showed a prevalence of 29.7% for pre-hypertension and 13.7% for hypertension^41^. Also, our findings showed that males were more pre-hypertensive and hypertensive than females despite having a lower BMI and WC which are measures of obesity. This finding concurs with a previous study done in Mthatha which showed similar results where the gender-specific prevalence of pre-hypertension/hypertension was higher in males (76.7%) compared to females (30.5%)^40^. These gender disparities may be linked to factors such as alcohol consumption, stress, biological metabolic differences, smoking, dietary intake and/or physical inactivity as previously reported^42^. Also, caffeine intake, drug abuse, awareness of hypertension and visiting of health facility could also be responsible for the gender difference in hypertensions previously reported^43^. These parameters were not used as exclusion criteria in the current study and may, therefore, play a role in the observed differences.

Recently, hypertension has been shown to be associated with increase gut permeability in humans^27^. Zonulin, a marker of impaired intestinal barrier, is one of the measurable physiological proteins that reflect gut permeability^34^. Indeed, gut permeability, as indicated by zonulin, has been associated with hypertension^44^. Findings in this study showed gut permeability to be associated with hypertension as zonulin level was higher in hypertensive subjects compared to normotensive subjects, especially in males. More so, there was a positive correlation between zonulin and systolic blood pressure in females. Gut permeability has previously been shown to be associated with obesity^34^. In this study, waist circumference, body mass index and visceral fat which are all measures of obesity were associated with zonulin and predicted gut permeability confirming previous findings which showed a relationship between zonulin and obesity in children^45^ and in adults^46^. These findings suggest that hypertension and obesity are associated with gut permeability.

Gut permeability is commonly associated with inflammation. This is as a result of alterations in the composition of microbiota (microbial dysbiosis) by high-fat diet and obesity which disrupts the tight junctions of the intestinal mucosa^5^. This leads to increase seepage of bacteria and bacterial components such as lipopolysaccharide (LPS) across the intestinal mucosa into the blood stream causing endotoxemia^9^. The high endotoxin level in blood leads to the release of pro-inflammatory cytokines including interleukin (IL)-1B, IL-12, IL-6 and TNF-α causing systemic inflammation^6^. Additionally, high circulating levels of pro-inflammatory cytokines also contribute to intestinal junction disassembly and gut permeability^9^. Adiponectin is an anti-inflammatory molecule which is expressed at high levels by lean healthy individuals^47^. Findings in this study showed no association between adiponectin and gut permeability. Also, reduced level of adiponectin has been observed in hypertension^48^ and obesity^49^. In this study, adiponectin was significantly lower in hypertensive subjects and negatively associated with SBP and DBP.

Also, adiponectin negatively associated with measures of obesity; WC, BMI and VF. More so, increased BMI and VC were shown to predict reduced adiponectin, thus, obesity to predict inflammation. These findings confirm previous reports which showed low levels of adiponectin to be associated with obesity^50^ and hypertension^51^. Adiponectin is known to possess vasculoprotective effects and therefore, hypoadiponectinaemia is considered an independent risk factor for endothelial dysfunction^52^. In this present study, adiponectin was not associated with markers of endothelial dysfunction.

Though previous studies have shown positive association between zonulin, blood pressure and body composition^33^, ^34^ as confirmed in this study, the relationship between gut permeability and endothelial dysfunction has not been addressed. Endothelial dysfunction is characterized by an imbalance in vasodilators and vasoconstrictrs of the endothelium^53^. Normally, the endothelium plays a protective role in blood vessels in reducing shear stress exerted by blood flow through the production of NO, a vasodilator that dilates the blood vessel reducing the stress^54^. Thus, reduced level of NO leads to endothelial dysfunction. Asymmetrical dimethylarginine (ADMA) on the other hand is a natural occurring product of protein metabolism found in the human circulatory system that acts as an inhibitor of NO synthesis. It acts by competing with arginine for nitric oxide synthase thereby inhibiting the synthesis of NO^24^. As such, increased ADMA levels are associated with reduced NO leading to endothelial dysfunction. Studies have shown an inverse relationship between ADMA and NO^55^, ^56^. Since endothelial dysfunction is associated with hypertension^57^, ADMA and low NO are potential risk factors for hypertension. Therefore, in hypertensive individuals, a high ADMA:NO ratio is expected with high circulating ADMA and low NO. Findings in this study showed no significant difference in ADMA concentrations between hypertensive and normotensive subjects. Contrary to expectations, NO was higher in hypertensive females compared to their normotensive counterparts. These findings do not suggest an association between hypertension and endothelial dysfunction which is contrary to already existing fact that hypertension is associated with endothelial dysfunction. This could be attributed to the age of the study participants who are young adults who were apparently healthy without any clinical manifestation of hypertension.

Gut permeability has been shown to be associated with inflammation^10^. During inflammation, pro-inflammatory cytokines such as TNF-α and IL-6 are released which contribute to vascular dysfunction^58^, ^59^. We therefore hypothesised that gut permeability may be associated with endothelial dysfunction implying that increased zonulin should be associated with reduced NO or increased ADMA levels which are characteristic of endothelial dysfunction. Findings in this study showed zonulin to be associated negatively with ADMA and positively with NO possibly indicating that gut permeability may improve endothelial function. These findings are contrary to our expectations because gut permeability which is an intestinal damage cannot promote vascular function. We therefore resolve to suggest that though gut permeability was not associated with endothelial dysfunction, it may not affect endothelial function. The possible explanation for this observation may be due to oxidative stress that results from inflammation in hypertensives^60^. Inflammatory cells generate free radicals and promote oxidative stress. Oxidative stress has been shown to up-regulate inducible NOS (iNOS) thereby leading to increase NO level^60^. This study is the first study to investigate the relationship between gut permeability and endothelial dysfunction in an African population of young adults. Therefore, further studies are needed to better establish these relationships and corroborate the observations made in this study. In this study, allergic pathologies were not assessed as an exclusion factor which may be a limitation since that can affect gut permeability.

## Conclusion

Gut permeability was associated with hypertension and measures of obesity but was not associated with endothelial dysfunction in a South African youth population. Adiponectin, an anti-inflammatory molecule was not associated with gut permeability and endothelial dysfunction. Hypertension was not associated with endothelial dysfunction although it was associated with reduced adiponectin. The association of hypertension as well as measures of obesity with gut permeability suggest that South African youths in this population may be at risk to diseases associated to gut permeability including inflammatory diseases (CID), allergic, autoimmune, metabolic diseases. This preliminary study serves as the basis for understanding the role of hypertension in gut permeability which is of public health concern. Further longitudinal studies may be needed to ascertain this finding.

## References

[1] Fasano A (2011). Zonulin and Its Regulation of Intestinal Barrier Function: The Biological Door to Inflammation, Autoimmunity, and Cancer. Physiology Review.

[2] Taylor WR, Takemiya K (2017). Hypertension Opens the Flood Gates to the Gut Microbiota. Circulation Research.

[3] Fine RL, Manfredo Vieira S (2020). Mechanisms and consequences of gut commensal translocation in chronic diseases. Gut Microbes.

[4] Hardin (2015). Increased Gut Permeability - Causes and Consequences. Arllegies and Your Gut.

[5] König J, Wells J, Cani PD (2016). Human intestinal barrier function in health and disease. Clinical and Translational Gastroenterology.

[6] Martin-Padura I, Lostaglio S, Schneemann M (1998). Junctional adhesion molecule, a novel member of the immunoglobulin superfamily that distributes at intercellular junctions and modulates monocyte transmigration. Journal of Cell Biology.

[7] Ikenouchi J, Furuse M, Furuse K, Sasaki H, Tsukita S, Tsukita S (2005). Tricellulin constitutes a novel barrier at tricellular contacts of epithelial cells. Journal of Cell Biology.

[8] Higashi T, Tokuda S, Kitajiri S (2013). Analysis of the ‘angulin’ proteins LSR, ILDR1 and ILDR2-tricellulin recruitment, epithelial barrier function and implication in deafness pathogenesis. Journal of Cell Science.

[9] Sturgeon C, Fasano A (2016). Zonulin, a regulator of epithelial and endothelial barrier functions, and its involvement in chronic inflammatory diseases. Tissue Barriers.

[10] Kim JH, Heo JS, Baek KS (2018). Zonulin level, a marker of intestinal permeability, is increased in association with liver enzymes in young adolescents. Clinica Chimica Acta.

[11] Ziccardi P, Nappo F, Giugliano G (2002). Reduction of inflammatory cytokine concentrations and improvement of endothelial functions in obese women after weight loss over one year. Circulation.

[12] Rask-Madsen C, Dominguez H, Ihlemann N, Hermann T, Kober L, Torp-Pedersen C (2003). Tumor necrosis factor-alpha inhibits insulin's stimulating effect on glucose uptake and endothelium-dependent vasodilation in humans. Circulation.

[13] Villela NR, Kramer-Aguiar LG, Bottino DA, Wiernsperger N, Bouskela E (2009). Metabolic disturbances linked to obesity: the role of impaired tissue perfusion. Arquivos Brasileiros de Endocrinologia & Metabologia.

[14] Adya R, Tan BK, Randeva HS (2015). Differential effects of leptin and adiponectin in endothelial angiogenesis. Journal of Diabetes Research.

[15] Durante W, Johnson FK, Johnson RA (2007). Arginase: a critical regulator of nitric oxide synthesis and vascular function. Clinical and Experimental Pharmacology and Physiology.

[16] Ohashi K, Ouchi N, Matsuzawa Y (2011). Adiponectin and hypertension. American Journal of Hypertension.

[17] Hwang HM, Lee JH, Min BS (2015). A novel arginase inhibitor derived from scutellavia indica restored endothelial function in ApoE-null mice fed a high-cholesterol diet. Journal of Pharmacology and Experimental Therapeutics.

[18] Willeit P, Freitag DF, Laukkanen JA (2015). Asymmetric dimethylarginine and cardiovascular risk: systemic review and meta-analysis of 22 prospective studies. Journal of American Heart Association.

[19] Zheng D, Liang Q, Zeng F (2015). Atorvastatin protects endothelium by decreasing asymmetric dimethylarginine in dyslipidemia rats. Lipids Health and Disease.

[20] Stühlinger MC, Abbasi F, Chu JW (2002). Relationship between insulin resistance and an endogenous nitric oxide synthase inhibitor. JAMA.

[21] Abbasi F, Asagmi T, Cooke JP (2001). Plasma concentrations of asymmetric dimethylarginine are increased in patients with type 2 diabetes mellitus. American Journal of Cardiology.

[22] Ito A, Egashira K, Narishige T, Muramatsu K, Takeshita A (2001). Renin-angiotensin system is involved in the mechanism of increased serum asymmetric dimethylarginine in essential hypertension. Japanese Circulation Journal.

[23] Perticone F, Sciacqua A, Maio R (2005). Asymmetric dimethylarginine, L-arginine, and endothelial dysfunction in essential hypertension. Journal of the American College of Cardiology.

[24] Sibal L, C Agarwal S, D Home P, H Boger R (2010). The role of asymmetric dimethylarginine (ADMA) in endothelial dysfunction and cardiovascular disease. Current Cardiology Review.

[25] Hawrelak JA, Myers SP (2004). The Causes of Intestinal Dysbiosis: A Review. Alternative Medical Review.

[26] Damms-Machado A, Louis S, Schnitzer A (2017). Gut permeability is related to body weight, fatty liver disease, and insulin resistance in obese individuals undergoing weight reduction. American Journal of Clinical Nutrition.

[27] Santisteban MM, Qi Y, Zubcevic J (2017). Hypertension-Linked Pathophysiological Alterations in the Gut. Circulation Research.

[28] Goel R, Kim S, Rigatto K, Shapiro B (2017). Abstract P134: Increased Gut Permeability and Dysbiosis in Patients With Pulmonary Arterial Hypertension. Hypertension.

[29] WHO (2019). Hypertension.

[30] Chopra HK, Ram CVS (2019). Recent guidelines for hypertension: a clarion call for blood pressure control in India. Circulation Research.

[31] Rayner B, Jones E, Veriava Y, Seedat YK (2019). South African Hypertension Society commentary on the American College of Cardiology/American Heart Association. Cardiovascular Journal of Africa.

[32] Morris-Paxton AA, Rheeder P, Ewing RMG, Ewing D (2018). Detection, referral and control of diabetes and hypertension in the rural Eastern Cape Province of South Africa by community health outreach workers in the rural primary healthcare project: Health in Every Hut. African Journal of Primary health Care & Family Medicine.

[33] Li J, Zhao F, Wang Y (2017). Gut microbiota dysbiosis contributes to the development of hypertension. Microbiome.

[34] Ohlsson B, Orho-Melander M, Nilsson P (2017). Higher levels of serum zonulin may rather be associated with increased risk of obesity and hyperlipidemia, than with gastrointestinal symptoms or disease manifestations. International Journal of Molecular Sciences.

[35] Stewart A, Marfell-Jones M, Olds T, Ridder H (2011). International Standards for Anthropometric Assessment.

[36] Giles TD, Materson BJ, Cohn JN, Kostis JB (2009). Definition and Classification of Hypertension: An Update. Journal of Clinical Hypertension.

[37] Miranda KM, Espey MG, Wink DA (2001). A rapid, simple spectrophotometric method for simultaneous detection of nitrate and nitrite. Nitric Oxide.

[38] Gyamfi D, Obirikorang C, Acheampong E (2018). Prevalence of pre-hypertension and hypertension and its related risk factors among undergraduate students in a Tertiary institution, Ghana. Alexandria Journal of Medicine.

[39] Tabrizi JS, Sadeghi-Bazargani H, Farahbakhsh M, Nikniaz L, Nikniaz Z (2016). Prevalence and associated factors of prehypertension and hypertension in Iranian Population: The Lifestyle Promotion Project (LPP). PloS One.

[40] Nkeh-Chungag BN, Mxhosa TH, Mgoduka PN (2015). Association of waist circumference and hip circumference with the presence of hypertension and pre-hypertension in young South African adults. African Health Sciences.

[41] Bhimma R, Naicker E, Gounden V, Nandlal L, Connolly C, Hariparshad S (2018). Prevalence of Primary Hypertension and Risk Factors in Grade XII Learners in KwaZulu-Natal, South Africa. International Journal of Hypertension.

[42] Singh S, Shankar R, Singh GP (2017). Prevalence and associated risk factors of hypertension: a cross-sectional study in urban Varanasi. International Journal of Hypertension.

[43] Agho KE, Osuagwu UL, Ezeh OK (2018). Gender differences in factors associated with prehypertension and hypertension in Nepal: A nationwide survey. PLoS One.

[44] Kim S, Goel R, Kumar A (2018). Imbalance of gut microbiome and intestinal epithelial barrier dysfunction in patients with high blood pressure. Clinical Sciences.

[45] Küme T, Acar S, Tuhan H (2017). The relationship between serum Zonulin level and clinical and laboratory parameters of childhood obesity. Journal of Clinical Research and Paediatric Endocrinology.

[46] Moreno-Navarrete JM, Sabater M, Ortega F, Ricart W, Fernandez-Real JM (2012). Circulating zonulin, a marker of intestinal permeability, is increased in association with obesity-associated insulin resistance. PloS One.

[47] Aprahamian TR, Sam F (2011). Adiponectin in Cardiovascular Inflammation and Obesity. International Journal of Inflammation.

[48] Ohashi K, Ouchi N, Matsuzawa Y (2011). Adiponectin and Hypertension. American Journal of Hypertension.

[49] Nigro E, Scudiero O, Monaco ML (2014). NewInsight into Adiponectin Role in Obesity and Obesity-Related Diseases. Biomed Research International.

[50] Balsan GA, Vieira JL, de Oliveira AM, Portal VL (2015). Relationship between adiponectin, obesity and insulin resistance. Review de Association Medicale Brasileiro.

[51] Iwashima Y, Katsuya T, Ishikawa K (2004). Hypoadiponectinemia is an independent risk factor for hypertension. Hypertension.

[52] Tan KC, Xu A, Chow WS (2004). Hypoadiponectinemia is associated with impaired endothelium-dependent vasodilation. Journal of Clinical Endocrinology and Metabolism.

[53] Rajendran P, Rengarajan T, Thangavel J, Nishigaki Y, Sakthisekaran D, Nishigaki I (2013). The vascular endothelium and human diseases. International Journal of Biological Sciences.

[54] Teixeira BC, Lopes AL, Macedo RCO (2014). Inflammatory markers, endothelial function and cardiovascular risk. Journal de Vascular Brasileiro.

[55] Triches CB, Mayer S, Quinto BMR, Batista MC, Zanella MT (2018). Association of endothelial dysfunction with cardiovascular risk factors and new-onset diabetes mellitus in patients with hypertension. Journal of Clinical Hypertension.

[56] Tain YL, Huang LT (2014). Restoration of asymmetric dimethylarginine-nitric oxide balance to prevent the development of hypertension. International Journal of Molecular Sciences.

[57] Gkaliagkousi E, Gavriilaki E, Triantafyllou A (2018). Asymmetric dimethylarginine levels are associated with augmentation index across naïve untreated patients with different hypertension phenotypes. Journal of Clinical Hypertension.

[58] Iantorno M, Campia U, Di Daniele N, Nisticò S, Forleo GB, Cardillo C, Tesauro M (2014). Obesity, inflammation and endothelial dysfunction. Journal of Biological Regulators & Homeostatic Agents.

[59] Savoia C, Sada L, Zezza L (2011). Vascular inflammation and endothelial dysfunction in experimental hypertension. International Journal of Hypertension.

[60] Zhen J, Lu H, Wang XQ, Vaziri ND, Zhou XJ (2008). Upregulation of Endothelial and Inducible Nitric Oxide Synthase Expression by Reactive Oxygen Species. American Journal of Hypertension.

